# First Reported Case of Lorazepam-Assisted Interview in a Young Indian Female Presenting with Dissociative Identity Disorder and Improvement in Symptoms after the Interview

**DOI:** 10.1155/2014/346939

**Published:** 2014-08-05

**Authors:** Raheel Mushtaq, Sheikh Shoib, Tasleem Arif, Tabindah Shah, Sahil Mushtaq

**Affiliations:** ^1^ECT Clinic, Postgraduate Department of Psychiatry, Government Medical College, Srinagar, Jammu and Kashmir 190010, India; ^2^Memory Clinic, Postgraduate Department of Psychiatry, Government Medical College, Srinagar 190010, India; ^3^Post Graduate Department of Dermatology, Government Medical College, Srinagar 190010, India; ^4^Government Medical College, Srinagar, Jammu and Kashmir 190010, India; ^5^Acharya Shri Chander College of Medical Sciences, Jammu, India

## Abstract

Dissociative identity disorder (DID) is one of the most fascinating disorders in psychiatry. The arduous search to reveal the obscurity of this disorder has led to colossal research in this area over the years. Although drug-assisted interviews are not widely used, they may be beneficial for some patients that do not respond to conventional treatments such as supportive psychotherapy or psychopharmacotherapy. Drug-assisted interviews facilitate recall of memories in promoting integration of dissociative information. We report a case of a 16-year-old female with dissociative identity disorder (DID) that was treated with lorazepam-assisted interview and there was rapid improvement in symptoms after the interview.

## 1. Introduction

Dissociation is a defensive mental process to exigency, a “shut-off mechanism” against severe anxiety and severe traumatic events, especially which involve serious threat to health or life. DSM IV TR classifies dissociative disorders into dissociative amnesia, dissociative fugue, dissociative identity disorder, depersonalization disorder, and dissociative disorder not otherwise specified [1–3]. The prevalence of dissociative amnesia (DA) in various epidemiological studies is found to be 1.8 to 7.3%. Sar et al. found the highest prevalence (7.3%) in Turkish women. In Ross's epidemiological study, DA was the most common (6%) dissociative disorder (DD). In other studies DA was the second common DD after dissociative disorders not otherwise specified (DDNOS). On the other hand dissociative fugue (DF) is quite rare [2]. In Ross's epidemiological study as well as in Johnson's epidemiological study, no cases of DF were found [3]. Sar's study found DF to be the least common DD and life time prevalence of DF was found to be 0.2% [3]. The prevalence of dissociative identity disorder (DID) in general population was found to be 1 to 3%. Various studies done in various countries found 1 to 5% of DID in inpatient psychiatry units [4]. The highest prevalence of DID (6–14%) was seen in emergency psychiatric departments [5].

In dissociative amnesia, there is sudden loss of memory for a variable period of time, without any central nervous system (CNS) disease, and that is not explained by normal forgetfulness and understanding of the patient that no memory loss has occurred [1, 6]. DID involves presence of two or more personalities and control behaviour and feeling of the dominant host with amnesia interfering with significant life events between the parts of the personality [7, 8]. In dissociative disorders, the disturbances in memory do not occur exclusively during the course of posttraumatic stress disorder, acute stress disorder, and somatisation disorder and also do not result from direct physiological effects of a substance, neurological disorder, or general medical conditions [1, 9, 10]. The various differential diagnoses of dissociative disorders are seizure disorder, substance intoxication, substance withdrawal, malingering, factitious disorder, psychotic disorders, and dementia. Dissociative amnesia occurs mostly in cases (without any history of psychiatric illness) involving sexual drives or physical injury to self [6, 9, 10]. DID occurs mostly in adolescents abused by caretakers in situations in which trauma occurs in the context of trusted relationship [7, 8]. The use of drug-assisted interviews in psychiatry dates back to 1938 and their usefulness has been explored in catatonia, confusion, conversion disorder, factitious disorder, mutism, dissociative amnesia, and DID [10–13]. Drug-assisted interviews are seen useful in patients of dissociative disorders that do not respond to pharmacological agents or psychological measures. Drug-assisted interview usefulness in dissociative disorders is by facilitating recall of memories in promoting integration of dissociative information [1, 12]. The various drugs used for drug-assisted interviews include amobarbital, thiopentone, diazepam, lorazepam, and midazolam. Although amobarbital is the most commonly used drug in the last 80 years, in recent years, benzodiazepines have begun to replace it to a large extent. The possible reasons for the decline in the use of amobarbital include the side effects of barbiturates, especially respiratory depression and absence of a specific antagonising agent [1, 11]. To the best of the knowledge of the investigator, there are just three case reports of dissociative amnesia in the literature on the use of lorazepam-assisted interviews [1]. However there are no case reports in the literature for the use of lorazepam-assisted interviews in DID, although there is a case report of a depressive patient, whose discovery of DID occurred by amobarbital interview [13]. We report a case of a 16-year-old Kashmiri (Indian) female with DID, that was treated with lorazepam-assisted interview and there was rapid improvement in symptoms after the interview.

## 2. Case Report

A 16-year-old unmarried Kashmiri (Indian) girl, student of the 10th class, nonsmoker, was referred by the neurologist for loss of memory for the last 5-month duration. Detailed history taken by the attendants revealed that she did not remember her name, did not recognise her relatives, including her parents, did not remember important events of her life like if she ever went to school, and even did not recognize her school friends. Whenever her parents would tell her to go to school, she would reply that she has never been to school and is an illiterate and does not know any arithmetic or any basic knowledge, that is expected for a 10th class student. She was also unable to play videogames on the computer, which she had played quite well for the last 5 years. There were symptoms of regressive behaviour in form of thumb sucking and talking like a child. She denied any history of traumatic event or history of sexual abuse in the past. The patient denied any history of head trauma or loss of consciousness in the past. There were no symptoms suggestive of seizure, manic episode, schizophrenia, anxiety, or organic disorders. She never drank alcohol or abused any psychoactive substances. Past medical, psychiatric, family, and personal histories revealed no significant findings. She was premorbidly introvert and had an avid interest in reading novels.

Mental state examination revealed a young cooperative girl who was appropriately dressed. She described her mood as euthymic. Thinking was goal directed with thoughts connected to each other. She had no delusions/hallucinations/derealisation/thought disorders. She was oriented to time, place, and person with intact attention and concentration at the time of the examination, performed serial sevens, digit span, and recalled 3 of 3 objects in 5 minutes. She had inability in remembering her name, information of the past (retrograde amnesia), could not tell her home address, personal biodata, and was also unable to recall events of her life, prior to the admission. Several questions of the examiner were associated with confabulations of embarrassment. She had no insight of her illness and appeared unconcerned for the same. Her physical examination was unremarkable. Neurological assessment and basic laboratory testing including electroencephalography (EEG), magnetic resonance imaging (MRI), and drug screening revealed no significant abnormalities. The Dissociative Experiences Scale (DES) was administered to the patient and she had a score of 50% on DES. The DES is an instrument used for screening dissociative disorders. High DES scores occur in chronic dissociative disorders like DID DF and DDNOS. Further score of 48% or above is indicative of diagnosis of DID [14]. Based on the longitudinal history, mental status examination (MSE) of the patient, and high score on DES (50%), a diagnosis of dissociative identity disorder was established as per DSM 5 [15] and patient was admitted to the hospital for further management.

Patient was engaged in psychotherapy by the clinical psychologist of the department. Psychoeducation was given to the patient and her parents, regarding this disorder. Sessions of psychotherapy were started by the clinical psychologist for the patient. However her memory did not recover. Therefore it was decided to use a drug-assisted interview for recovery of her memory. Informed consent was obtained from the patient as well as her parents. Two psychiatrists participated as interviewers and an anaesthesiologist monitored and recorded her clinical state during the interview. The interview was performed according to previously described protocol [1, 11]. During the drug-assisted interview, the patient was able to recall memories that precipitated the incident. In the interview, she stated that she was sexually abused by her cousin, who was 12 years her elder. She further stated that sexual abuse started 6 months ago, when her cousin would come to their house to help her in studies. She told that she was too shy and afraid to report about the same to her parents. The interview lasted approximately one hour. During this time, she expressed her anger and venom towards her cousin. As soon as the drug-assisted interview ended, the patient fell into a deep sleep. The day after her interview, she was able to recall portions of what she had said, during the drug-assisted interview. As several days passed, she appeared more depressed and reported experiencing severe anxiety. The use of supportive psychotherapy, reassurance, and oral clonazepam 0.5 mg twice a day was added. After her depressed mood and anxiety had improved, she was discharged on the 21st day.

On two-month follow-up, patient had responded well to supportive psychotherapy. She did not remember the 6 months of her life prior to hospital admission; however she remembered all her events prior to the 6-month amnesia. On 6-month follow-up, she was able to appear for her final examinations. She however passed in none of the three subjects she appeared for. She reported no further periods of amnesia or regressive behaviour. Further follow-up of the patient could not be done, as we lost the patient subsequently.

## 3. Discussion

Freud in his psychoanalytic theory suggested that dissociative disorders represent an unconscious attempt to avoid an unbearable conflict [6]. The common characteristics of all dissociative disorders are a disruption in the normal centralizing functions of identity, memory, or awareness, with features ranging from simple retrograde amnesia to full DID [7]. The psychoanalytic theory also views DID as an adaptive response important for sustaining attachment to caretakers in circumstances in which trauma or traumatic events occur in the background of a trusted relationship [8]. We reported a case of a 16-year-old girl of dissociative identity disorder of 6-month duration, which did not respond to any psychotherapy or pharmacotherapy. The change in identity included inability to remember her name and relatives, not remembering her personal biodata, and change in behaviour (regressive behaviour). The regressive behaviour occurred in form of thumb sucking and talking like a child. The regression to a younger state was not consistent with simple DA or transient childish fugue identity in DA, as there was continuous change in personality and not remembering her biodata for 6 months. Further higher scores on DES (50%) and trauma (sexual abuse) occurring in the context of a trusted relationship (patient's cousin) confirmed the diagnosis of dissociative identity disorder, a type of chronic dissociative disorder. As there was no improvement in patient's symptoms with psychotherapy or pharmacotherapy, a drug-assisted interview was sought. Barbiturates were used as part of the standard treatment for dissociative amnesia [16]. However in recent years benzodiazepines have replaced amobarbital for drug-assisted interviews [17]. The reason for this could be due to lesser side effects and decreased interactions of drugs with benzodiazepines, compared to barbiturates [16, 18]. Further the results for use of lorazepam in drug-assisted interviews are similar to that of barbiturates [9, 11]. With lorazepam-assisted interviews, we recalled repressed memories that were associated with undesirable conflicts and adverse life events. In keeping with the psychoanalytic theory, the sexual abuse committed on a 16-year-old may have been so unbearable that amnesia and change in identity resulted. Repression of memory explains the protection of the emotional pain, that emerges from severe anxiety and disturbing traumatic events. Thus DID occurred as an unconscious attempt to avoid the conflict. The drug-assisted interview worked like a chemical buffer against emotional pain and allowed the patient to verbalize about the undesirable and traumatic events [12]. Recently it has been seen that stress can facilitate encoding of memories by the hippocampus, by release of glucocorticoids. Repeated severe stress does the opposite, by diverting the hippocampal encoding mechanism and increasing functions of the amygdale [7]. Stress can also have a direct effect on medial temporal–diencephalic system. When traumatic memories are recalled, the medial temporal–diencephalic system is inhibited. Further lesion in the medial temporal and the diencephalic areas of the brain also causes an amnesic syndrome [1, 6, 9]. Amnesic syndrome is sustained thus by release of stress hormones and causing functional dissociation of the front temporal regions [18, 19]. Amnesia is also associated with increased activity of N-methyl-D-aspartate (NMDA) receptors [19]. Therefore we assume that, in drug-assisted interview, lorazepam inhibited prefrontal activity and decreased NMDA receptor activity.

The claims of recovered repressed memories of childhood sexual abuse represent a striking phenomenon in the mental health. It has been speculated that repression may be one method used by individuals to cope with traumatic memories, by pushing them out of awareness (perhaps as an adaptation via psychogenic amnesia). Thus drug-assisted interview recalled repressed memories that were associated with emotional pain and thus allowed the patient to verbalize about the undesirable and traumatic events. As patient reported mild depression and severe anxiety after the interview, the use of reassurance, supportive psychotherapy, and oral clonazepam 0.5 mg twice a day was sought to resolve these symptoms. In conclusion, use of lorazepam in drug-assisted interviews is very effective and safe for resolving dissociative amnesia.

## 4. Conclusion

Drug-assisted interviews are useful in patients of DID that do not respond to pharmacological or psychological measures. Drug-assisted interview usefulness in dissociative disorders facilitates recall of memories and promotes integration of dissociative information. Over the years, benzodiazepines have replaced other drugs for drug-assisted interviews because of lesser side effects, good results, and decreased interactions of other drugs with it.
